# Editorial: Role of angiotensin-converting enzyme in myeloid immune functions

**DOI:** 10.3389/fphys.2023.1297995

**Published:** 2023-09-28

**Authors:** DuoYao Cao, Suguru Saito

**Affiliations:** Department of Biomedical Sciences, Cedars-Sinai Medical Center, Los Angeles, CA, United States

**Keywords:** ACE - angiotensin-converting enzyme, myeloid cells, macrophage, immune response, chronic disease

Angiotensin-converting enzyme (ACE) is best known for its effects on blood pressure regulation due to its ability to generate the potent vasoconstrictor angiotensin II. Besides cleaving angiotensin I, ACE degrades many substrates, including bradykinin, substance P, N-acetyl-seryl-aspartyl-lysyl-proline (AcSDKP), enkephalins, neurotensin, amyloid β peptide, and others (Bernstein et al., 2013). ACE is composed of two homologous catalytic domains termed the N- and C-domains, each domain has a specific catalytic preference (Bernstein et al., 2013). Recent research findings indicate that ACE impacts various physiological processes, including normal renal development, male reproductive function, hematopoiesis, aspects of the immune response, and metabolic functions in chronic diseases (Bernstein et al., 2018; Cao et al., 2020a; Cao et al., 2020b; Cao et al., 2021; Cao et al., 2022; Cao et al., 2023).

In this Research Topic, Danziger et al. discussed that myeloid ACE affects Alzheimer’s disease (AD). ACE has demonstrated the ability to degrade Aβ42, a neurotoxic peptide that caused AD, suggesting a potential neuroprotective function. Research in murine models has indicated that augmenting ACE expression within specific immune cells holds promise for reducing neuropathology and enhancing cognitive function in AD. This review may provide insights into the potential for therapeutic approaches in Alzheimer’s disease based on ACE’s natural capabilities.

The Renin-angiotensin-aldosterone system (RAAS) is a pivotal regulator of blood pressure, primarily influencing renal sodium reabsorption, vascular tone, and sympathetic output. Additionally, it is closely linked to the blood pressure response triggered by changes in salt intake. In the findings by Ertuglu et al., high sodium exposure *in vitro* resulted in the downregulation of renin, renin binding protein, and renin receptor expression. However, no significant alterations were detected when examining the genes of the renin-angiotensin system in response to dietary salt loading and depletion *in vivo*. Notably, salt-sensitive individuals tended to have lower plasma renin levels and a diminished response to the salt loading/depletion challenge. These results demonstrate that short-term dietary salt changes do not impact RAAS expression in myeloid immune cells.

A healthy body’s immune system must address potential threats from both external and internal sources. When disturbances occur, such as blood pressure or electrolyte balance disruptions, the RAS system perceives danger signals, leading to increased blood pressure. Blood vessel endothelial cells release abundant ACE into the bloodstream to regulate blood pressure and support immune cells circulating in the system, such as neutrophils and monocytes. These cells stand ready to combat infections and tumors. ACE as one of the most vital and extensively studied peptidases in the RAS, plays an important role in myeloid immune response. However, in recent years, evidence shows ACE has participated in many immune responses, especially in myeloid immune response, such as bacterial clearance, anti-tumor, and reduced atherosclerotic plaque (Khan et al., 2019; Cao et al., 2021; Cao et al., 2023).

Due to the effects of ACE in regulating blood pressure and other cardiovascular diseases, ACE inhibitors (ACEi) are extensively employed in the treatment of hypertension, heart failure, and various cardiovascular conditions. This raises concerns for patients who rely on ACEis for long-term blood pressure management and cardiovascular health. Intriguingly, recent investigations have suggested that ACE deficiency might compromise the immune functions of neutrophils and macrophages, particularly in the context of bacterial infections and tumor progression (Khan et al., 2019; Cao et al., 2020a; Cao et al., 2021; Cao et al., 2022). Conversely, myeloid cells with elevated ACE levels may enhance immune response and metabolic functions (Cao et al., 2020b; Cao et al., 2023). These studies suggest that ACE activity within myeloid cells is pivotal in modulating the immune response. However, our comprehension of the impact of ACE inhibition on myeloid cells during acute and chronic inflammation in patients receiving ACEi treatment (e.g., individuals with hypertension, heart failure, and diabetes) remains limited. Therefore, there is a compelling need for a more precise exploration of pharmacological treatments related to ACE inhibition, demanding further consideration.

This Research Topic has the potential to draw increased interest in ACE functions and to encourage more individuals to dedicate their efforts to exploring ACE in immune cells.

## References

[B1] Bernstein K. E. Khan Z. Giani J. F. Cao D. Y. Bernstein E. A. Shen X. Z. (2018). Angiotensin-converting enzyme in innate and adaptive immunity. Nat. Rev. Nephrol. 14, 325–336. 10.1038/nrneph.2018.15 29578208 PMC6192041

[B2] Bernstein K. E. Ong F. S. Blackwell W. L. Shah K. H. Giani J. F. Gonzalez-Villalobos R. A. (2013). A modern understanding of the traditional and nontraditional biological functions of angiotensin-converting enzyme. Pharmacol. Rev. 65, 1–46. 10.1124/pr.112.006809 23257181 PMC3565918

[B3] Cao D. Khan Z. Li X. Saito S. Bernstein E. A. Victor A. R. (2023). Macrophage angiotensin-converting enzyme reduces atherosclerosis by increasing peroxisome proliferator-activated receptor α and fundamentally changing lipid metabolism. Cardiovasc Res. 119, 1825–1841. 10.1093/cvr/cvad082 37225143 PMC10681664

[B4] Cao D. Veiras L. Ahmed F. Shibata T. Bernstein E. A. Okwan-Duodu D. (2022). The non-cardiovascular actions of ACE. Peptides 152, 170769. 10.1016/j.peptides.2022.170769 35182689 PMC10405936

[B5] Cao D. Y. Giani J. F. Veiras L. C. Bernstein E. A. Okwan-Duodu D. Ahmed F. (2021). An ACE inhibitor reduces bactericidal activity of human neutrophils *in vitro* and impairs mouse neutrophil activity *in vivo* . Sci. Transl. Med. 13, eabj2138. 10.1126/scitranslmed.abj2138 34321319 PMC10370421

[B6] Cao D. Y. Saito S. Veiras L. C. Okwan-Duodu D. Bernstein E. A. Giani J. F. (2020a). Role of angiotensin-converting enzyme in myeloid cell immune responses. Cell Mol. Biol. Lett. 25, 31. 10.1186/s11658-020-00225-w 32508938 PMC7249647

[B7] Cao D. Y. Spivia W. R. Veiras L. C. Khan Z. Peng Z. Jones A. E. (2020b). ACE overexpression in myeloid cells increases oxidative metabolism and cellular ATP. J. Biol. Chem. 295, 1369–1384. 10.1074/jbc.RA119.011244 31871049 PMC6996878

[B8] Khan Z. Cao D. Y. Giani J. F. Bernstein E. A. Veiras L. C. Fuchs S. (2019). Overexpression of the C-domain of angiotensin-converting enzyme reduces melanoma growth by stimulating M1 macrophage polarization. J. Biol. Chem. 294, 4368–4380. 10.1074/jbc.RA118.006275 30670595 PMC6433053

